# How Ah Receptor Ligand Specificity Became Important in Understanding Its Physiological Function

**DOI:** 10.3390/ijms21249614

**Published:** 2020-12-17

**Authors:** Iain A. Murray, Gary H. Perdew

**Affiliations:** Department of Veterinary and Biomedical Sciences, Center for Molecular Toxicology and Carcinogenesis, The Pennsylvania State University, University Park, PA 16802, USA; iam1@psu.edu

**Keywords:** AHR, Ah receptor, agonist, tryptophan, cytokines, microbiome, indole

## Abstract

Increasingly, the aryl hydrocarbon receptor (AHR) is being recognized as a sensor for endogenous and pseudo-endogenous metabolites, and in particular microbiota and host generated tryptophan metabolites. One proposed explanation for this is the role of the AHR in innate immune signaling within barrier tissues in response to the presence of microorganisms. A number of cytokine/chemokine genes exhibit a combinatorial increase in transcription upon toll-like receptors and AHR activation, supporting this concept. The AHR also plays a role in the enhanced differentiation of intestinal and dermal epithelium leading to improved barrier function. Importantly, from an evolutionary perspective many of these tryptophan metabolites exhibit greater activation potential for the human AHR when compared to the rodent AHR. These observations underscore the importance of the AHR in barrier tissues and may lead to pharmacologic therapeutic intervention.

## 1. Introduction

The seminal identification of the Ah receptor (AHR) as the mediator of dioxin toxicity classified the AHR as a xenobiotic receptor [1,2,3]. Purification, cloning and subsequent studies highlighted this toxic mediator function of the AHR with regard to a class of polycyclic and halogenated aryl hydrocarbon compounds, many of which represent persistent organic pollutants (POPS) [4,5,6,7,8]. From numerous toxicological studies arose a dichotomy, an evolutionary conserved protein is a mediator for numerous POP chemicals that are largely anthropogenic and have only existed since the dawn of the industrial age. Clearly, the existence and evolutionary persistence of the AHR is not a consequence of a need to mount a toxic response to such anthropogenic POP chemicals. Evidently, the AHR must perform some physiological function in the absence of exposure to these chemicals.

**Figure 1:**
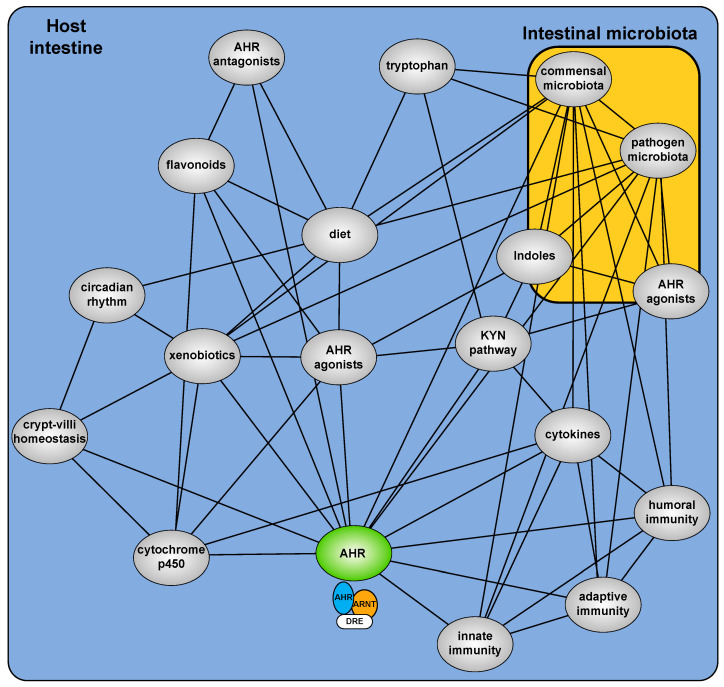
Overview of the AHR interactome and how it translates into effects on epithelium and innate immune surveillance.

The development of *Ahr^−/−^* mice provided a critical starting point to examine the hitherto cryptic physiologic function of the AHR [9,10,11]. These mice, in the absence of exogenous exposure, exhibit deleterious phenotypes arising from an implied insensitivity to endogenous (rather than xenobiotic) AHR ligands [12]. Multiple sources of xenobiotic independent physiological AHR activity are now described including dietary factors, microbial metabolites, endogenous metabolites and the combined impact of secondary signaling processes (Figure 1). It is increasingly evident that physiological AHR activity is nuanced, involving a complex cooperative/competing ”interactome” and repositions the AHR from a toxicant mediator to an important sensor of physiological homeostasis.

## 2. Endogenous Ligand/Activators

The search for, and validation of, high-affinity, high-specificity endogenous AHR ligands has been notoriously challenging. Due to the extreme hydrophobicity/high affinity of dioxin relative to most of the putative endogenous ligands, competition-binding assays with radiolabeled dioxin are technically challenging [13]. Notwithstanding, numerous and chemically distinct endogenously generated compounds are represented in the literature as endogenous ligands. Typically, these candidate endogenous ligands have been inferred from their capacity to induce expression of CYP1A1, which exhibits a remarkable dependency and sensitivity to AHR activation.

A principal contender as an endogenous AHR ligand was 6-formylindolo [3,2b] carbazole (FICZ), a photo-oxidation product of tryptophan [14,15]. FICZ fulfills the criteria, exhibiting high-affinity/specificity (similar to 2,3,7,8-tetrachlorodibenzo-*p*-dioxin, TCDD), high potency with regard to CYP1A1 induction and is detectable in vivo [16,17,18,19]. Additionally, FICZ activity has been revealed to be transient, by virtue of it being a metabolic substrate for CYP1A1, thus establishing a FICZ/AHR/CYP1A1 autoregulatory loop, consistent with an endogenous ligand [20]. More recently, a mechanism for UV-independent FICZ generation has been proposed, involving deamination/oxidative rearrangement of indole derivatives of microbial origin [21]. The production of FICZ, through UV-mediated photo-oxidation however, is largely spontaneous and unregulated and thus somewhat incompatible with highly regulated AHR physiology. In addition, the concept that most metabolites of physiologic importance are made through an enzymatic process, the exception being small molecules required in the diet (e.g., certain vitamins).

Although, tryptophan itself does exhibit AHR ligand activity, its metabolic products have been revealed as an important source of physiological AHR activity. Circulating tryptophan levels are largely regulated through hepatic tryptophan dioxygenase (TDO) and peripheral indolamine dioxygenase (IDO) activities, respectively. Both TDO and IDO catalyze the same rate-limiting conversion of tryptophan to kynurenine, which is then further metabolized along a kynurenine pathway (KP) to generate physiologically important substrates, principally NAD [22,23]. An AHR activity screen identified the KP metabolites, kynurenic acid and xanthurenic acid, as AHR activators [24]. Subsequently, radiolabeled ligand competition studies revealed kynurenic acid as a direct AHR ligand that exhibits greater selectivity towards human AHR compared to rodent. Importantly, both kynurenic acid binding and capacity to induce AHR activity (1 µM) lie within the detectable physiological concentrations of kynurenic acid. Indeed, such concentrations have been identified within bile and pancreatic secretions, suggesting that kynurenic acid may play a role in establishing basal hepatic CYP1A1 and stimulating intestinal AHR activity [25]. Although included as part of the KP-metabolite screen, kynurenine failed to stimulate AHR activity, indeed additional studies fail to demonstrate kynurenine-mediated AHR activation [24]. Further studies however identified kynurenine as an AHR ligand [26]. The discrepancy between these studies may involve the cell lines and kynurenine concentrations utilized since cell-dependent effects have been reported. Moreover, AHR activation by kynurenine appears to be relatively weak, requiring high micromolar concentrations. Recent evidence has cast doubt upon the veracity of kynurenine as a bone fide AHR ligand, rather in vitro/modeling data is presented suggesting that kynurenine is a proligand which can undergo condensation reactions to generate so-called trace-extended aromatic condensation products (TEACOPs) with greatly enhanced AHR activation potential [27]. Whether such TEACOPs are detectable in vivo remains to be established. Nonetheless, the activation of AHR by kynurenine (or potential condensation products) has been shown to play a significant role in determining immunological fate and activity under pathological conditions, particularly cancer, where kynurenine levels are significantly increased [28,29,30]. Under such conditions, AHR activation by kynurenine promotes intrinsic AHR-dependent tumor cell survival combined with reshaping of the immunological milieu to invoke an immune tolerant environment within solid tumors [24,31,32,33]. Although the endogenous relevance of KP-mediated AHR activation has not been fully established, the pathological immune tolerance of this interaction suggests the normative function of KP-AHR is to provide physiological tolerance where immunological stress is apparent, i.e., within barrier tissues that interface with the external environment. Such tissues are continually exposed to antigenic stimuli, e.g., from the microbiota, and require immunological sensitivity within the context of tolerance to restrict excessive inflammatory/immunological activation. Recently, a TDO/IDO-independent route to kynurenic acid production and AHR activation has been identified [34]. An IL4-inducible component (IL4L1) stimulates kynurenic acid production from indole-3-pyruvic acid to promote tumor tolerance. Intriguingly, IL4 provides tolerance within the intestinal tract and indole-3-pyruvic acid is produced by the microbiota, additionally kynurenic acid and AHR activity are both evident within the intestine [35,36]. It remains to be established whether such a mechanism provides a tolerogenic component to the commensal microbiota.

While KP-dependent processes have gained prominence as an endogenous source of AHR activators, other endogenous ligands have been described. Analysis of tissue extracts identified 2-(1′H-indole-3′-carbonyl)-thiazole-4-carboxylic acid (ITE) as a candidate ligand [37,38]. ITE clearly activates the AHR, its mode of extraction upon initial identification (acid hydrolysis of crude lung tissue) however, casts some doubt on its physiological relevance. Although the AHR exhibits promiscuity with regard to ligand binding, a perceived common (toxicological) feature has been that AHR activators present as low molecular weight polycyclic planar compounds. A number of putative endogenous activators do not adhere to this model. Certain arachidonic acid metabolites, including 5,6-diHETE, 12R-HETE and a number of prostaglandins, have been demonstrated to elicit AHR activation [39,40,41]. However, the physiological significance of AHR activation by these metabolites remains to be established. In a similar, atypical, fashion, modest AHR activation has been observed with modified LDL isolated from sera undergoing vascular shear stress. Direct AHR activation by LDL seems implausible, thus it has been proposed that AHR activation is in response to LDL-mediated kynurenine production [42,43]. Such data is consistent with reports indicating an important role for the AHR within the vasculature. With particular relevance to vascular physiology, 7-ketocholesterol has also been proposed as an endogenous modulator of AHR function [44]. Uniquely amongst the list of endogenous modulators, 7-ketocholesterol exhibits AHR antagonism.

## 3. Pseudo-Endogenous Ligands/Activators

An increasing number of pseudo-endogenous (i.e., not produced by the organism but still intrinsically present within it) AHR activators have been described. Such activators are derived principally from dietary precursors or the microbiota. Numerous plants and isolated constituents consumed by humans have demonstrable AHR activity (either activation or inhibition) in vitro [45,46]. However, the physiological relevance of many of these dietary constituents in isolation is questionable. First, many exhibit weak AHR activity in isolation; second, and perhaps more importantly, most are present in trace amounts on a weight basis. Such low abundance renders dietary consumption, even if ingested regularly, extremely impractical. It may be argued that while any given dietary constituent may not be present in sufficient abundance to physiologically activate AHR signaling, the compilation and cumulative action of many weak activators may provide a physiological level of activation. This notion of cumulative action is consistent with the xenobiotic receptor function of AHR. The greatest potential for exposure to toxins is through the diet, indeed many plants contain toxic secondary metabolites that require metabolic clearance. Promiscuous activation of the AHR by numerous weak phytochemicals likely provides a metabolic shield to guard against potentially harmful effects from ingestion of toxic co-phytochemicals.

Two classes of dietary constituents stand out as being sufficiently abundant and/or potent to represent significant dietary modulators of AHR activity, glucobrassicins and flavonoids. Vegetables of the genus *Brassica* (Broccoli, Brussel sprouts, etc.) contain significant quantities of glucobrassicins, which upon digestion, liberate indole-3-carbinol (I3C), 3,3′-diindoylmethane and indole-3-acetonitrile, each of which exhibits weak AHR activity [47]. However, in the acid-rich environment of the stomach, I3C undergoes acid condensation to form indolo [3,2b]carbazole (ICZ). ICZ represents a potent AHR activator, indeed in rodent studies, a diet comprising 15% (*w/w*) broccoli is sufficient to stimulate significant intestinal and to a lesser extent, systemic AHR activity [48]. Furthermore, such stimulation has been demonstrated to mitigate intestinal pathology. The flavonoids, comprising thousands of distinct three-ring polyphenols, represent a class of widely distributed phytochemicals. Numerous flavonoids exhibit AHR activity, either weakly stimulating or inhibiting AHR-mediated transcription [13,49,50,51,52,53]. Many of the flavonoids described as modulators of AHR function have proposed health benefits, however it is unclear if such benefits are AHR dependent given the wide-ranging biological activities attributed to flavonoids.

The resident microbiota inhabiting mucosal surfaces (gastrointestinal, respiratory, reproductive) and skin have gained extensive attention as modulators of host physiology. Indeed, a reciprocal interaction between the microbiota and the AHR has been demonstrated [48,54,55,56,57,58]. This interaction is believed to be facilitated through microbial generation of AHR activators and subsequent AHR-mediated host signaling, which in turn regulates the microbiota. The number of putative microbiota-dependent AHR activators is extensive [56]. Although not exclusively, many of these microbial activators are low molecular weight indoles derived from microbial tryptophan metabolism [58]. In the intestine, indole represents the most abundant microbial tryptophan metabolite-exhibiting AHR activity [36,59]. At concentrations detected in rodent cecal contents and human feces, indole provides saturating human AHR agonist activity using in vitro assays. In vivo however, AHR activity is not saturating despite high indole levels. This is presumably due to partitioning into fecal matter and that fecal concentrations do not represent intracellular indole concentrations. Secondly, the saturating activity is a consequence of abundance rather than potency, for human AHR indole exhibits weak agonist activity whereas for mouse AHR agonist activity is negligible [59]. Microbial conversion of tryptophan to numerous derivatives such as; indole-3-acetic acid, indole-3-pyruvate, indole-3-lactate, 3-methyl indole, indole-3-aldehyde, tryptamine and tryptanthrin, provides additional avenues of AHR modulation [60]. The microbiota has also been revealed as a source of the tryptophan metabolite, kynurenic acid [61]. Recently, additional microbiota-dependent metabolites have been described as AHR modulators, including 2, 8-dihydroxquinoline, and 2-oxindole [36]. Activation of AHR signaling in response to microbial metabolites not directly derived from tryptophan have also been reported [56].

Clearly, the microbiota does not generate these compounds for the purpose of activating host AHR. Many of these microbial AHR activators have been shown to exhibit quorum-sensing activity, i.e., they serve as intermicrobe communication signals to regulate microbial growth within a particular niche [59,62,63,64]. Speculatively, it would appear that the AHR acquired microbial metabolite binding capacity and that this adaptation coevolved with AHR-mediated regulation of the immune system to promote a symbiotic environment beneficial to both host and microbe. In this regard, the AHR behaves as a microbial sensor akin to host pattern recognition receptors, e.g., toll-like receptors [65]. The role of the AHR as a sensor/regulator of the intestinal microbiota has been highlighted with rodent studies demonstrating that *Ahr−/−* and *Ahr^−/+^* littermates undergo significant shifts in microbial community structure despite exposure to an identical maternal microbiome [55]. In essence, *Ahr^−/−^* animals have a restricted surveillance of the microbiota and thus are less able to immunologically constrain microbial dysbiosis [65].

In addition to tryptophan-derived microbial AHR ligands, microbial metabolism facilitates bioactivation of dietary phytochemicals into AHR modulators. Most phytochemicals exist as poorly absorbed glycoside conjugates and, in this form, are unable to modulate AHR. The microbiota harbors extensive glycosidase activity and can deconjugate phytochemicals thus allowing absorption and AHR modulation. An example is provided by ellagic acid which exhibits no AHR activity, however, microbial metabolism of ellagic acid generates the AHR antagonist urolithin A [66]. Importantly, urolithins have been shown to have anti-inflammatory properties which may involve AHR modulation. It is likely that microbial metabolism of phytochemicals exerts a major role in dictating physiological AHR activation and the health-promoting effects of numerous phytochemicals. In contrast, microbial bioactivation of AHR modulators has also been shown to be deleterious. Microbial metabolism of dietary tryptophan generates significant quantities of indole and its derivatives, a number of which exhibit AHR activity. Although largely eliminated in feces, systemic indole concentrations are detectable in the micromolar range. Conversion of indole to indoxyl by hepatic CYP2E1 and sulfation by SULTs generates indoxyl sulfate, which exhibits AHR agonist activity with enhanced specificity towards the human AHR [67]. Indoxyl sulfate represents a potent uremic toxin, promoting vascular defects associated with chronic kidney disease [68]. It has been proposed that persistent AHR activation by indoxyl sulfate in the context of kidney disease may contribute to enhanced inflammatory signaling [67].

## 4. CYP1A1 Competitive Substrates/Inhibitors

As mentioned previously, many endogenous and pseudo-endogenous AHR activators have been inferred from their capacity to induce CYP1A1 expression. However, it has become apparent that this criterion is insufficient to validate AHR ligand status. A number of endogenous AHR activators, including flavonoids, arachidonic acid metabolites and certain tryptophan metabolites have been demonstrated to be metabolized by CYP1A1 [16,20,69,70,71]. Thus, it can be envisioned that, in the context of these CYP1A1-labile AHR activators, any compound that may or may not itself bind AHR but presents as a competitive CYP1A1 substrate or inhibitor, will effectively increase AHR activator bioavailability and manifest as an AHR activator. Evidence supporting the feedback from CYP1A1 to influence AHR activity is provided from studies examining tissue-specific constitutive CYP1A1 expression. Lung tissue exhibits high *Cyp1a1* expression relative to other tissues, presumably as a consequence of high AHR ligand bioavailability. Lung extracts from *Ahr−/−* mice (essentially *Cyp1a1* negative) exhibit a significant capacity to induce AHR activity in an AHR-sensitive heterologous reporter system [72]. This data suggests the presence of CYP1A1-labile AHR ligand activity in the lung. Interestingly, serotonin does not bind the AHR but still stimulates intestinal AHR activity by virtue of being a CYP1A1 substrate and decreasing metabolic clearance of CYP1A1-labile ligands [73]. Further evidence is provided from studies demonstrating that forced intestinal CYP1A1 overexpression generates a quasi-AHR deficiency leading to diminished intestinal immune function [71]. Conversely, exposure to CYP1A1 inhibitors increases AHR activity, elevating Th17-mediated surveillance and modulation of enteric neuronal activity [74,75]. Similarly, tissue-specific (intestine), conditional ablation of Ah receptor nuclear translocator (ARNT) expression, effectively restricts intestinal CYP1A1 metabolism of dietary/microbial AHR ligands, thus increasing bioavailability and systemic AHR activity [72].

Many of the dietary, endogenous and pseudo-endogenous AHR modulators exhibit weak activity either as a consequence of weak affinity for AHR and/or due to rapid metabolic inactivation. However, the relative lack of potency may represent an important aspect of AHR physiology. Toxicological studies indicate that persistent potent AHR activation (e.g., with POPs) leads to numerous toxic outcomes whilst transient, pulsatile potent activation (e.g., with FICZ) does not. Such observations suggest that ligand persistence rather than potency is the dominant factor that segregates AHR-mediated toxicity from AHR-dependent physiology. Such a conclusion suggests that the core pharmacokinetic concepts of absorption, distribution, metabolism and excretion likely play a significant role in dictating the normative physiological role of AHR. In many pathological states, AHR activity is enhanced and this may be attributed to increased ligand availability and a concomitant switch from pulsatile to more persistent AHR activation. While genetic studies demonstrate that ablation of AHR activity is also deleterious, such observations suggest that physiologically, AHR functions as a homeostatic regulator and that low potency but continuous modulation of AHR activity by complex mixtures of weak agonists/antagonists and indirect modulators, subtly regulates an expansive AHR-dependent transcriptome to ultimately maintain homeostasis.

## 5. Evolution

Since its initial identification, studies have highlighted that, despite a remarkable conservation of overall AHR function within vertebrates, the AHR exhibits significant differences in ligand responsiveness across, and indeed within, species [76,77,78]. Many of these studies have focused on differential sensitivity to toxic outcomes associated with POP exposure. However, the characterization of numerous sources of dietary, endogenous and pseudo-endogenous routes to AHR activation have allowed an examination of AHR ligand selectivity within this context and how this may relate to sensitivity to toxic xenobiotics. Given the physiological action of dietary, endogenous and pseudo-endogenous AHR ligands, such studies may provide greater insight into normative AHR function.

Historically, the human AHR has been considered less responsive when compared to rodent AHR within the context of xenobiotic POP exposure. Such differences can be attributable to amino acid differences within the AHR ligand binding domain [79]. Intriguingly, the reduction in POP sensitivity does not appear to correlate with an overall reduction in sensitivity to physiological AHR activators. Studies have revealed that human AHR retains equivalent or enhanced sensitivity towards indirubin, kynurenic acid, indole, 2-oxindole, indoxyl sulfate, 2, 8-dihydroxyquinoline, when compared to rodent AHR [24,36,59,67,80]. Such observations raise the question as to the selective pressures that may have prompted such a divergence in human AHR ligand selectivity. In a recent comparative study, it was noted that the amino acid sequence encoding the AHR ligand binding domain of modern humans is invariant, i.e., that in representative genomes spanning multiple ethnicities and groups the sequence is uniformly conserved. However, this conserved sequence differs at a single residue (human valine 381) when compared to gorilla, chimpanzee and new world monkey AHR (alanine 381) [81]. Importantly, each of these primates exhibits a greater selectivity towards xenobiotic ligands compared to human AHR, whilst exhibiting similar activation by a panel of endogenous ligands. Such data suggests that divergence to the modern, less xenobiotic sensitive human sequence occurred after the evolutionary branching of their respective lineages. Comparison between the AHR ligand binding sequence and that of extant Neanderthal genomes revealed that these close relatives harbor the alanine 381 variant, which confers greater sensitivity to xenobiotic ligands. It has been speculated that the divergence of this sensitivity determinant may have been coincident with a pivotal societal event common to both homo lineages, i.e., the utilization of fire. Incomplete combustion of organic material generates large quantities of particulate matter rich in polycyclic hydrocarbons (PAH) that can potently activate the AHR leading to PAH toxicity. Thus, *H. neanderthalensis* were likely more sensitive than their *H. sapiens* counterparts, to fire-induced PAH toxicities. Perhaps more importantly than the development of longer-term PAH toxicity, is the potential for acute exacerbation of copathologies, common to both lineages, e.g., respiratory and intestinal infections. It is therefore likely that diminished PAH sensitivity conferred a selective advantage to the *H. sapiens* lineage. Recently, this conclusion has been contradicted with evidence suggesting that *H. neanderthalensis* and *H. sapiens* AHR exhibit equivalent sensitivity to environmental pollutants despite significant profound intrinsic differences in PAH binding affinity between the species [82]. It is suggested that differences in relative *H. neanderthalensis* and *H. sapiens* AHR expression levels within the dubiously termed “homologous” context of forced expression in human AHR expressing Hela cells effectively negates the impact of intrinsic ligand affinity. Whilst expression levels may influence AHR activity to some degree, it has been established that aryl hydrocarbon-mediated toxicity across species correlates with AHR binding affinity [83]. Additionally, it is difficult to rationalize (as far as one can ascribe directionality to evolutionary processes) a presumed selective pressure that fixed a single mutation in the AHR ligand binding domain that profoundly reduces sensitivity to toxins, if such a reduction does not confer any advantage. Further evidence supporting acute evolutionary changes in AHR-dependent signaling within the context of xenobiotic exposure has been observed in fish species [84]. Importantly, decreased PAH sensitivity of human AHR did not occur at the expense of a change in responsiveness to a panel of dietary, endogenous or pseudo-endogenous ligands, thus further highlighting the importance of such non-xenobiotic AHR activation to overall fitness.

## 6. Ah Receptor and Cytokines/Chemokines

**Figure 2:**
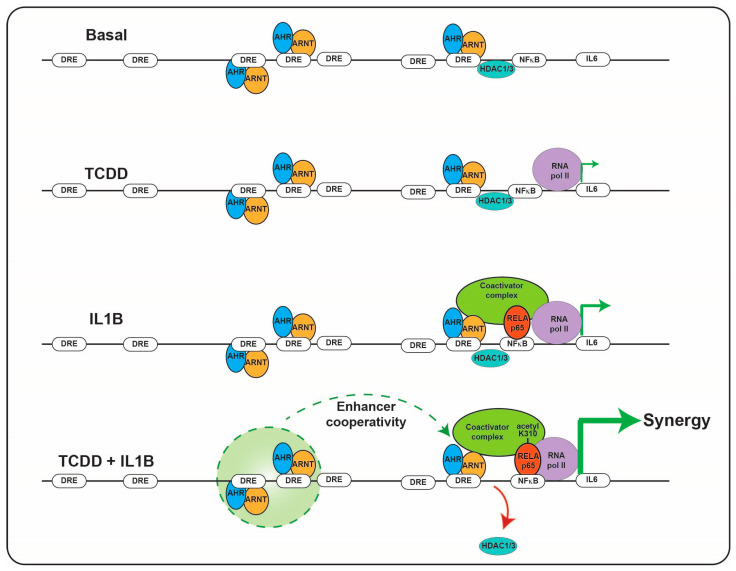
Combinatorial regulation of IL6 promoter by the AHR and NFkB. The presence of AHR/ARNT and NFkB on the IL6 promoter results in HDAC1/3 dismissal and subsequent hyperacetylation of NFkB resulting in synergistic induction of IL6 transcription.

**Figure 3:**
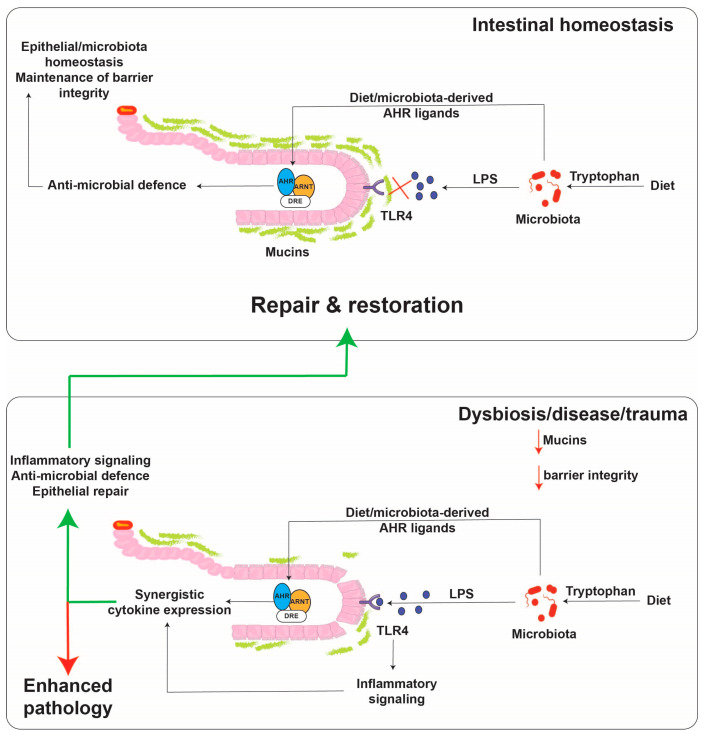
Proposed model for cytokine regulation by the AHR and TLR4 in the intestinal tract. Small molecules are capable of diffusing across the mucus lining and binding to the AHR, while TLR4 ligands, such as lipopolysaccharide as much larger molecules, do not diffuse across the mucus lining except upon a breach. The presence of both activators leads to robust cytokine/chemokine production. The activated AHR then aids in the repair response to the breach.

Immunotoxicities have long been established as hallmark toxic endpoints arising from xenobiotic AHR ligand exposure. Many of these toxicities are attributable to aberrant expression of cytokines and chemokines, leading to inappropriate immune cell lineage commitment, trafficking and function. Such immune dysfunction is likely a consequence of persistent modulation of immune regulators that physiologically are regulated by dietary, endogenous and pseudo-endogenous AHR ligands in a less potent, more transient, homeostatic fashion. An emerging concept is that physiological rather than toxicological cytokine/chemokine modulation by the AHR contributes to epithelial barrier function through the sensing of AHR ligands generated in situ by the microbiota or by the host, in response to the microbiota. A number of cytokine/chemokine genes have been shown to be regulated in a costimulatory fashion in the context of an AHR ligand combined with immunological stimulation, e.g., proinflammatory cytokines or toll-like receptor ligation. Perhaps the cytokine/chemokine gene promoter that has been studied the most extensively in terms of such a costimulatory mechanism is *IL6* [85]. The *IL6* 3 kb upstream regulatory sequence harbors a number of noncanonical, imperfect dioxin response elements that facilitate constitutive AHR binding along with HDAC1/3 without stimulating transcription. Exposure to AHR ligands stimulates low level transcription of IL6. The IL6 promoter also harbors NFkB response elements that provide IL6 transcription in response to IL1B or TLR4 stimulation. Costimulation with AHR ligands and IL1B facilitates a marked synergistic induction of IL6 [86]. The proposed mechanism of synergistic induction is through AHR-mediated displacement of HDAC1/3 from the *Il6* promoter and subsequent acetylation of p65, this has been shown to lead to increased gene transcription (Figure 2) [87]. In the context of potent and persistent xenobiotic (or endogenous, e.g., kynurenine) AHR activation combined with inflammatory signaling, such IL6 synergy likely contributes to the sustained proinflammatory oncogenic phenotype of many tumors. It has been proposed that this costimulatory mode of cytokine induction “hijacked” by tumors may have a nonpathological physiological function with particular relevance to barrier tissues. Barrier tissues are continually exposed to the microbiota and their products, including potent inflammatory stimulants, e.g., pathogen-associated molecular patterns (PAMPs) such as lipopolysaccharides, flagellin and DNA, yet intact barrier tissues are somewhat tolerant to stimulation in order to restrict excessive inflammation. Such tolerance, is in part, mediated by the physical barrier itself, which prevents direct access of PAMPs to the immune system. As described previously, the barrier associated microbiota provides a rich source of diverse but weak AHR ligands. In contrast to high-molecular weight PAMPs, these AHR ligands are of lower molecular weight and are barrier permeable, as evidenced by their systemic detection. Thus, in this context of an intact barrier, there is a surveillance level of IL6 expression provided by AHR and restricted immune stimulation by PAMPs. In this environment, the IL6 promoter could be considered “primed”. However, should a breakdown in barrier integrity occur, PAMPs would have direct access to epithelial cells and the immune system resulting in NFkB activation. Thus, with the IL6 promoter “primed”, the costimulatory action of AHR ligands and PAMPs would become evident and facilitate a rapid synergistic induction of IL6, allowing establishment of an appropriate immune response and subsequent repair mechanisms (Figure 3). In addition, the activation of NFkB can lead to a direct increase in AHR expression, potentially amplifying the AHR activation component of this costimulatory mechanism [88].

The list of cytokine/chemokine genes that are directly regulated by the AHR continues to grow and now includes, *Ccl20*, *Cxcl5*, *Il1b*, *Il33*, *IL10* and *Il27* [89,90,91,92,93]. These observations have led to labeling this subset of cytokines/chemokines within the battery of AHR target genes as “xenokines” [90].

Another study that further supports the importance of AHR in the regulation of chemokines is the ability of *P. aeruginosa* pigments to enhance *Cxcl1/5* expression in pneumocytes in an AHR-dependent manner [56].

**Figure 4:**
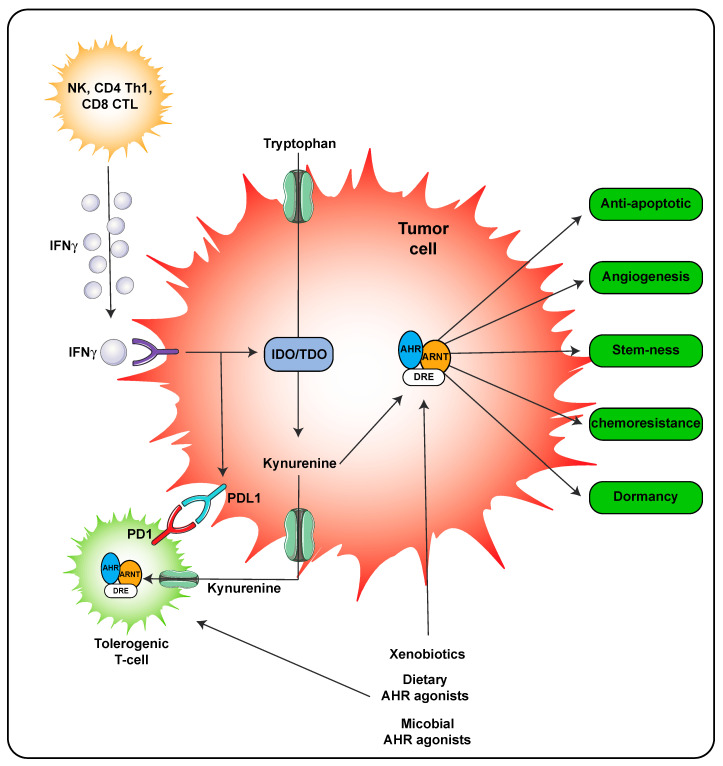
AHR activity within the tumor microenvironment. AHR activation in both the tumor cell and immune cells occurs in the presence of kynurenine leading to tolerogenic T-cells. Activation of the AHR in tumor cells also leads to numerous processes that lead to tumor survival and outgrowth. The following abbreviations are defined as; NK, natural killer cells; CD4, cluster of differentiation 4; Th1, T helper 1; CD8, cluster of differentiation 8; PD-1, programmed cell death protein 1; IFNγ, interferon gamma.

A second mechanism of AHR ligand production is through host metabolism. In the inflammatory environment during a viral or some bacterial infections, IFNγ is secreted by immune cells leading to significant expression of IDO that in turn metabolizes tryptophan to kynurenine, which is a weak AHR agonist. However, the concentrations of kynurenine can reach relatively high concentrations locally suggesting that the AHR would likely be activated (Figure 4). Kynurenine is also found in high concentrations in tumors and through activation of the AHR can lead to adaptive immune suppression [28,32]. Importantly, the use of AHR antagonist can attenuate resistance to immune checkpoint inhibitors in IDO/TDO expressing tumors and thus effectively work in combination with immune checkpoint inhibitors as a treatment regimen [31].

In an LPS acute exposure mouse model a cytokine storm occurs that, along with other responses, leads to death. Since the AHR can participate in innate immune signaling, attenuating AHR activity may be beneficial. A selective AHR ligand SGA360 that exhibits the ability to enhance cytoplasmic retention and thus inhibit AHR-mediated transcriptional activity was tested in an LPS acute phase model [94]. Coexposure to LPS and SGA360 led to a 40% decrease in lethality in *Ahr^+/+^* mice compared to LPS treatment alone. In contrast, SGA360 had no effect on lethality levels in *Ahr−/−* mice. Examination of the expression of genes directly involved in inflammation were examined in a lower dose LPS exposure model and SGA360 was capable of significantly attenuating *Il6*, *Il1b*, *Tnfa* expression in lung, liver and kidney tissues. In a monosodium urate crystal peritonitis infiltration model, SGA360 also repressed immune cell infiltration into the peritoneum [94]. These studies suggest that the AHR is an active participant in acute inflammation and may be a useful target to consider in the treatment of acute inflammatory diseases such as septic shock or gout. The mechanism of how the AHR is activated in these studies is not known. However, peritoneal extracts from LPS exposed mice revealed an increase in AHR activation potential in a cell-based assay.

## 7. Barrier Function and AHR

The AHR is highly expressed in barrier epithelial cells and its role in cellular differentiation is likely to lead to enhanced barrier function. Studies in *Ahr−/−* mice reveal enhanced trans-epidermal water loss compared to *Ahr^+/+^* mice [95]. Epidermal differentiation is impaired in cultured *Ahr−/−* mouse keratinocytes or upon treatment of *Ahr^+/+^* keratinocytes with an AHR antagonist [96]. In addition, AHR antagonism in human keratinocyte air interface cultures inhibits barrier protein expression, epidermal stratification and stratum corneum formation. Interestingly, coal tar has long been used to treat skin diseases, and the mechanism of therapeutic activity is at least in part through AHR activation [97]. A number of reports have documented the ability of the AHR to increase expression of skin barrier proteins such as filaggrin, loricrin and involucrin either directly or through increased expression of OVO-like 1 [96,98,99,100,101]. Interestingly, AHR activation in the skin can lead to enhanced production of IL22 expressing T cells, which can potentiate atopic dermatitis [102,103]. These observations would suggest that the excessive presence of AHR agonist could be detrimental. However, in the context of a bacterial or viral infection, the production of AHR ligands could lead to an enhanced or repressed innate immune response in a context specific manner [29,94,104]. The role of AHR activation in the gut epithelium appears to be Wnt-βb-catenin signaling in intestinal epithelial cells, which will also play a role in barrier function [105,106,107]. The AHR has been directly implicated in maintaining intestinal differentiation. For example, AHR mediated induction of IL22 during an injury is thought to enhance barrier function through stimulating epithelial cell migration, thus increasing barrier function [108,109]. The AHR is capable of regulating intestinal stem cell proliferation, through modulating tight junction integrity through regulation of junction protein expression, especially during toxic insults [110,111,112,113]. Specific deletion of the AHR from intestinal epithelium results in a compromised response to *C. rodentium* infection [105], underscoring the importance of epithelial AHR expression in intestinal homeostasis and protection.

## 8. AHR, Wound Healing, and Cancer

One aspect of barrier homeostasis is responding to a wound, the AHR appears to influence wound healing and tissue remodeling through modulation of local chemokine expression and altering gene expression in neighboring epithelial or fibroblast cells [110,114,115,116]. The ability of the AHR to directly regulate Slug expression further strengthens the concept that the AHR directly modulates wound healing [117]. The ability of a combined inflammatory signaling and AHR activation to induce growth factors may also play a role in wound healing, although this has not been directly examined [118]. Importantly, in AHR null mice wound closure re-epithelialization is enhanced, although the effect of various AHR ligands has not been tested [119]. Clearly, additional in vivo studies and human clinical studies are needed to assess the utility of modulating AHR in wound closure. Often the tumor microenvironment is compared to a wound in that many of the same cell types are present and there is a heightened state of inflammation due to the presence of infiltrating immune cells. The role of the AHR within the tumor microenvironment has been recently reviewed [120]. The AHR plays a role in both innate and adaptive immune responses and is now considered a possible therapeutic target in cancer treatments (Figure 4) [31,51,121,122]. In addition to the role of the AHR in innate immune signaling, there is another major mechanistic story that has developed. The ability of IFNγ secreted from T cells to induce indoleamine dioxygenase in tumor cells leads to the production of kynurenine from tryptophan. Enhanced kynurenine levels within the tumor microenvironment stimulates AHR activation within T-cells resulting in elevated PD1 expression, which in combination with tumor cell intrinsic PDL1 expression introduce immune checkpoints leading to immune tolerance [123]. This process can be delayed through the use of AHR antagonist and improves the efficacy of anti-PD1 immunotherapy [31]. Development of AHR antagonists for human therapeutics is currently being explored.

## 9. AHR Activation during a Stress Response

AHR ligands are likely elevated in a yeast or bacterial infection where there is an abundance of tryptophan [124]. In addition, AHR ligands are present in many plants that also contain potentially toxic metabolites, such as isothiocyanates. Both of these exposures could elicit a localized stress response in the affected tissue. Thus, it follows that the AHR might participate in the cellular response to stress as a xenobiotic sensor. One form of stress is oxidative stress that leads to DNA damage. ICZ protects established intestinal cell lines from DNA damage in an AHR dependent manner [125]. The AHR is also capable of regulating IL22, which has been shown to protect the intestinal stem cell compartment from genotoxic stress [109,126]. Another aspect of mediating maximal DNA repair is to stall cell division to allow the DNA repair systems time to complete repairs prior to cell division. Indeed, AHR activation in the intestinal epithelium has been shown to attenuate cell proliferation [105,107]. These observations add to the overall picture of how the AHR plays a central role in response to external stimuli in barrier tissues.

## 10. Summary

The AHR is a xenobiotic sensor that is activated by a diverse array of high-affinity low molecular weight, largely anthropogenic, ligands which have provided enormous insight into its biological role(s) within a toxicological context. More recently, attention has turned to intrinsic modes of AHR activation arising from tryptophan metabolites, especially within the immune compartment. Yet another source of ligands can be found in foods, such as cruciferous vegetables containing the precursor indole-3-carbinol. Fourth and perhaps the most abundant source is through metabolism of tryptophan by commensal bacteria in the gut. Such low-level pulsatile, yet relatively weak (in comparison to xenobiotic ligands) activation of AHR by dietary, endogenous and pseudo-endogenous ligands provides nuanced modulation of critical physiological functions essential to homeostasis and health, i.e., maintenance of epithelial barrier integrity combined with the sensing and modulation of the microbial landscape through appropriate immune activation within generally immune tolerant environments such as the gastrointestinal tract. Data suggests that such modes of physiological AHR activation have been retained despite the evolutionary recent adaptation of human AHR towards diminished xenobiotic sensitivity. This retention highlights the physiological importance of dynamic, weak AHR activation with regards to overall human health.

## Abbreviations

AHR	Aryl hydrocarbon receptor
ARNT	Ah receptor nuclear translocator
KP	kynurenine pathway
POPs	persistent organic pollutants
